# Blood pressure control with active ultrafiltration measures and without antihypertensives is essential for survival in hemodiafiltration and hemodialysis programs for patients with CKD: a prospective observational study

**DOI:** 10.1186/s12882-025-03948-0

**Published:** 2025-01-17

**Authors:** Franklin Geovany Mora-Bravo, Pamela Tatiana Morales Torres, Nelson Rojas Campoverde, Guillermina Lucía Blum Carcelen, Juan Cristobal Santacruz Mancheno, Ángel Cristóbal Santacruz Tipanta, Hector Perez-Grovas, Willan Patricio Robles Abarca

**Affiliations:** 1 Pafram Hemodiafiltration Unit, Complementary Health Network, Sucúa, Morona Santiago Ecuador; 2 Hemodialysis Unit of the Renal Foundation of Ecuador in Guayaquil, Guayaquil, Ecuador; 3 Menydial Kidney Clinic, Quito, Ecuador; 4 Nephrology Service, Instituto Nacional de Cardiología Ignacio Chavez, Mexico City, México; 5 Medical career, Faculty of Health Sciences, Universidad Técnica de Ambato, Ambato, Ecuador

**Keywords:** Antihypertensives, Hemodiafiltration, Mortality, Point of Care Dry Weight, Survival

## Abstract

**Background:**

High blood pressure is a prevalent condition in patients with chronic kidney disease on hemodialysis. Adequate control of high blood pressure is essential to reducing deaths in this group. The present study aimed to observe mortality prospectively in a group of patients in hemodialysis and hemodiafiltration programs in whom the use of antihypertensives was optimized with the point-of-care dry weight (POC-DW) technique.

**Methods:**

The present observational, prospective study was carried out at the Pafram hemodiafiltration unit in Morona Santiago, Ecuador, and the hemodialysis unit of the Fundación Renal del Ecuador in Guayaquil, Ecuador, from August 2019 to December 2023. Patients who were receiving hemodiafiltration were included. Weight was optimized with POC-DW for eight weeks. In Group 1, patients whose use of antihypertensive drugs was not required to control systolic blood pressure with a value less than 150 mmHg predialysis, less than 130 mmHg postdialysis, and a peridialytic blood pressure (defined as post-HD minus pre-HD SBP) between 0 and − 20 mmHg were analyzed. In Group 2, patients who required antihypertensive drugs for not meeting the aims of systolic blood pressure were included. The variables included clinical, demographic, mortality, description of the treatment, and routine laboratory tests in dialysis programs. The sample was nonprobabilistic. Survival analysis was performed for the study groups. The log-rank test (Mantel-Cox) was used for survival comparisons.

**Results:**

The study included 106 patients. Optimal blood pressure control without antihypertensive treatment was achieved in 52 patients (49.1%) (Group 1). In 54 patients (50.9%), antihypertensive agents were required (Group 2). There was more significant mortality in the group that received antihypertensives: 11 patients in group 1 (21.2%) versus 25 patients in group 2 (46.3%) (*P* = 0.005). Survival was more significant in group 1, with an HR of 2.2163 (1.125–4.158) (*P* = 0.0243).

**Conclusion:**

In hemodiafiltration and hemodialysis programs, blood pressure control with active ultrafiltration measures and without using antihypertensives is essential for survival in patients with CKD.

**Supplementary Information:**

The online version contains supplementary material available at 10.1186/s12882-025-03948-0.

## Introduction

High blood pressure is a prevalent condition in patients with chronic kidney disease (CKD). Between 60% and 90% of patients on hemodialysis have it, and it is associated with hypervolemia [1]. Hypertension is associated with hypervolemia due to excess sodium intake and poor dry weight adjustment. Other factors, such as vasodilation and sympathetic control, occur in 5 to 10% of patients on hemodialysis, accounting for 95 to 90% of the hypervolemia cases.

Hypervolemia is an independent risk factor for fatal outcomes in patients with CKD on hemodialysis programs. Volume overload in hemodialysis patients is associated with hypertension and cardiac dysfunction and is a significant risk factor for cardiovascular and all-cause mortality in this population. Hypervolemia is also associated with an inflammatory state in hemodialysis patients [2].

Optimal volume management involves three key components: accurately estimating volume status, correcting extracellular fluid overload, and preventing intradialytic instability.

A gold standard for assessing volume status is needed for an accurate estimate. Clinical examination has insufficient sensitivity and specificity; for example, the Tassin method of probing and dry weight was no longer associated with improved survival. Consequently, fluids are suggested to be managed even more actively, incorporating several objective measurements of volume status together [3]. Tools to assist in objectively measuring extracellular fluid volume require further validation. However, bioimpedance spectroscopy is arguably the most widely used method for subjectively quantifying fluid distributions in body compartments and produces reliable and reproducible results. Lung ultrasound provides reliable estimates of extravascular water in the lung, a critical parameter of the central circulation that primarily reflects left ventricular end-diastolic pressure [4]. However, these measurements still need to be applied in clinical practice.

Recommendations for volume control include avoiding rapid correction of hypervolemia due to the risk of precipitating intradialysis hypotension and hypoperfusion of vital organs, including the heart, brain, liver, intestine, and kidneys [5]. To maximize cardiovascular tolerance, fluid elimination in volume-expanded HD patients should be gradual and distributed over a sufficiently long period [4].

Observational studies consistently show worse survival in patients with predialysis systolic blood pressure < 140/90 mmHg. However, such studies are likely confounded by low blood pressure due to CVD and other comorbidities [6]. Several treatment alternatives to reduce blood pressure in these patients do not require additional drug therapy (e.g., long slow hemodialysis, short daily hemodialysis, nocturnal hemodialysis, dietary salt, fluid restriction, or reducing the sodium concentration in the dialysate). These parameters provide good blood pressure monitoring, even for patients with previously diagnosed hypertension [7]. Until additional data are available, we should treat hypertension during hemodialysis by actively pursuing euvolemia through dry weight catheterization and reducing excess salt [7].

On the other hand, the prescription of antihypertensives in normotensive patients is a problem, and a response needs to be identified. For example, a study demonstrated that a high plasma refilling rate at the beginning of hemodialysis is associated with intradialytic hypotension. This finding suggested that hypervolemia (high refilling) is a possible factor associated with intradialytic hypotension independent of the ultrafiltration refilling rate [8]; however, the effects of antihypertensive drugs have not been considered. The study hypothesizes that there is more remarkable survival in patients with CKD whose hypertension can be controlled without antihypertensives and with constant dry weight reduction measures to optimize ultrafiltration. The present study aimed to observe mortality prospectively in a group of patients in hemodialysis and hemodiafiltration programs in whom the use of antihypertensives was optimized with the point of care dry weight (POC-DW) technique.

## Materials and methods

### Study design

The present study was observational. The source is prospective.

### Scenery

The study was carried out in the Pafram Hemodiafiltration Unit in the city of Sucúa (900 m above sea level), Morona Santiago-Ecuador, and in the Hemodialysis Unit of the Fundación Renal del Ecuador in Guayaquil (4 m above sea level), Ecuador. The observation period was from August 1, 2019, to December 31, 2023.

### Participants

Adult patients with a diagnosis of stage 5-d chronic renal failure in renal function replacement programs with hemodialysis or hemodiafiltration were included. Only patients who survived to baseline and had no missing covariates were included. The baseline survival time for this study was two months.

### Study groups

This study used an active ultrafiltration strategy for eight weeks and intradialytic monitoring by a nephrologist who conducted ultrafiltration throughout the treatment. This ultrafiltration maneuver, called POC-DW, clearly differentiated the two groups.

Group 1: included patients whose dry weight was optimized at eight weeks and whose use of antihypertensive drugs was not required to control predialysis systolic blood pressure less than 150 mmHg, whose post-dialysis systolic blood pressure was less than 130 mmHg, and whose peridialytic blood pressure (defined as post-HD minus pre-HD SBP) was between 0 and − 20 mmHg.

Group 2: Patients who, after the 8-week dry weight optimization period, required the use of antihypertensives, had a predialysis blood pressure greater than 150 mmHg, a post-dialysis systolic blood pressure greater than 130 mmHg, and a peridialytic blood pressure (defined as post-HD minus pre-HD SBP) less than − 20 mmHg.

### Variables

The variables were age, sex, survival time in months, mortality, type and number of antihypertensive drugs used, comorbidities, smoking status, cause of chronic kidney disease, type of access, presence of diabetic blindness or significant vascular retinal lesion, presence of vascular amputation of limbs, presence of active cancer, treatment modality (hemodialysis-hemodiafiltration), and vintage. The average survival in the last month before data censoring was calculated for treatment variables such as pre and post-treatment weight, ultrafiltration, intradialytic weight gain, replacement volume, and effective blood flow (QB). The previous quartile of treatment survival averages for laboratory tests before data censoring were calculated. The laboratory parameters used were hemoglobin, lymphocytes, saturation of transferrin, ferritin, glucose, urea, creatinine, cholesterol, triglycerides, albumin, TGP, alkaline phosphatase, sodium, potassium, calcium, phosphate, and PTH.

### Data sources/measurements

The source was direct; an electronic form was used to fill out the data collected during the study period. Intention-to-treat (ITT) data were preferred compared to per-protocol data. The information was confidential; no personal data were included in identifying the study subjects. The patients signed informed consent to participate in the study.

### Procedures

#### Point-of-care Dry Weight (POC-DW)

**Fig. 1:**
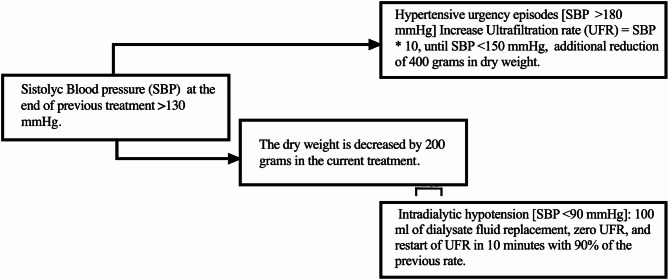
Point-of-care dry weight. Point of care dry weight (POC-DW) algorithm

The initial treatment weight was established for all patients, and a decision algorithm for ultrafiltration was established (Fig. 1). With this algorithm, the dry weight was corrected continuously for eight weeks. Intradialysis hypertensive urgency episodes with systolic pressures greater than 180 mmHg were treated with an increase in the ultrafiltration rate with the following formula: [ultrafiltration rate (ml/hour) = systolic blood pressure * 10] until the decrease in blood pressure was less than 150 mmHg, with which an additional reduction in the calculation of the dry weight of 400 g was proposed. Intradialytic hypotension was treated with 100 ml of dialysate fluid replacement, zero ultrafiltration rate, and restart of ultrafiltration in 10 min with 90% of the previous rate. A nephrologist remained constantly in the hemodiafiltration room during the 4 h of each treatment to guide prescriptions and treatment. At the established time of 8 weeks, after the period of optimization of dry weight, if the patient presented a predialysis systolic blood pressure greater than 150 mmHg and a postdialysis systolic blood pressure greater than 130 mmHg, there was a peridialytic blood pressure (defined as post-HD minus pre-HD SBP) less than − 20 mmHg [9], antihypertensive treatment was started. The systolic blood pressure records present the average data of the last month of treatment for the patient’s survival or censoring.

### Biases

To avoid interviewer, information, and memory biases, the leading researcher always maintained the data with a guide and records approved in the research protocol. Observation and selection bias were avoided by applying participant selection criteria. Two researchers independently analyzed each record in duplicate, and the variables were registered in the database once their agreement was verified.

### Study size

The sample was nonprobabilistic and census-type, where all possible cases from the study period were included.

### Quantitative variables

Descriptive statistics were used. The results are expressed as frequencies (categorical variables) and means (numerical variables). Categorical data are presented in proportions.

### Statistical analysis

Survival analysis was performed for the study groups. The log-rank test (Mantel–Cox) was used for survival comparisons. Screening events included renal transplantation, modality change, and change in treatment location due to a change in address or study end. The statistical package used was SPSS 27.0 (IBM Corp., 2020). IBM SPSS Statistics for Windows, Version 27.0. Armonk, NY: IBM Corp.).

## Results

### Participants

**Fig. 2:**
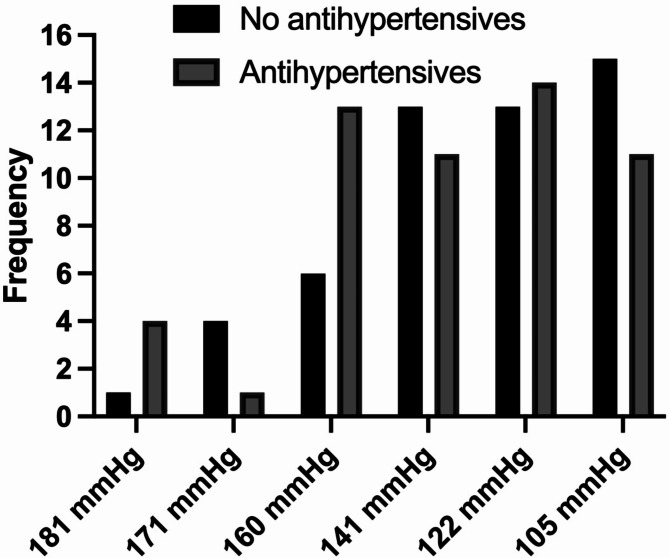
Study participants classified by blood pressure sextiles

A total of 13 patients were eliminated due to early death before two months of survival at the beginning of the hemodialysis or hemodiafiltration program. The study included 106 patients. In 52 patients, optimal blood pressure control was achieved without antihypertensive drugs (49.1%) (Group 1). In 54 patients (50.9%), antihypertensives were required (Group 2). The 95% confidence interval for a proportion for Group 1 was 39.58 − 58.62%. Figure 2 shows the distribution of the frequency of arterial hypertension by sextile according to the average arterial pressure obtained in the last month of follow-up or censorship.

### Characteristics of the study groups

There were 34 women (65.4%) in group 1 and 19 women (35.2%) in group 2 (*P* = 0.002). A total of 80.2% of all patients underwent hemodiafiltration treatments. There were 47/52 patients (90.4%) in hemodiafiltration in group 1 and 38/54 patients (70.4%) in group 2 (*P* < 0.01). There were 27 new incident cases in group 1 (51.9%) and 28 in group 2 (51.9%). The prevalence period in group 1 was 28.4 ± 37.9 months, and in group 2, it was 55.6 ± 43.3 months. The average age was 50.7 years in Group 1 and 59.2 years in Group 2 (*P* = 0.018). The two groups had similar body mass indices. The antihypertensive group had greater height and body weight. In group 2, there were 37 patients (68.5%) treated with amlodipine, 24 (44.4%) with atenolol/carvedilol, 19 (35.2%) with losartan and 7 (13%) with other antihypertensives. In group 2, 25 patients (48.2%) used one antihypertensive drug, 20 patients (37%) used two antihypertensive medications, and eight patients (14.8%) used three antihypertensive drugs. Table 1 presents the data related to hemodialysis or hemodiafiltration treatment.

**Table 1:** Variables of the study group

	Group 1 Without Antihypertensives *N* = 52	Group 2 With Antihypertensives *n* = 54	*P*
Age (Years)	50.7 ± 21.1	59.2 ± 14.4	0.018
BMI (kg/m2)	24.92 ± 4.1	26.18 ± 4.9	0.160
**Treatment data (average of the last month)**			
Pretreatment weight (kg)	60.44 ± 14.02	66.75 ± 15.83	0.032
Ultrafiltration (L)	3.022 ± 0.900	2.753 ± 0.873	0.122
Post-treatment weight (kg)	57.60 ± 13.70	64.09 ± 15.75	0.026
IDWG (%)	5.44 ± 1.76	4.45 ± 1.57	0.003
Systolic blood pressure pre-treatment (mmHg)	137.8 ± 23.8	143.7 ± 24.5	0.209
HDF volume (L)	21.92 ± 3.29	22.90 ± 6.29	0.360
HD time (min)	216.0 ± 13.4	219.4 ± 13.3	0.420
HDF time (min)	217.0 ± 10.3	213.0 ± 15.7	0.415
HD Effective blood rate -QB (Ml/min)	372.0 ± 21.7	379.4 ± 16.9	0.434
HDF Effective blood rate -QB (Ml/min)	445.7 ± 45.0	426.3 ± 56.9	0.084
Erythropoietin dosage (U/Kg/Week)	47.37 ± 51.27	47.84 ± 57.59	0.990
**Laboratory tests (average of the last 3 months)**			
Hemoglobin g/dl	10.66 ± 1.82	10.64 ± 2.05	0.950
Lymphocytes (u/ul)	1.844 ± 0.523	1.865 ± 0.523	0.859
Transferrin saturation (%)	25.31 ± 13.49	32.75 ± 17.23	0.009
Ferritin (mg/dl)	258.9 ± 415.1	388.5 ± 472.3	0.151
Glucose (Mg/dl)	131.9 ± 75.1	174.2 ± 107.1	0.021
Urea (mg/dl)	95.5 ± 35.9	120.3 ± 53.1	0.006
Creatinine (mg/dl)	8.00 ± 2.70	8.22 ± 4.25	0.753
Cholesterol (mg/dl)	189.6 ± 44.5	178.3 ± 41.7	0.238
Triglycerides (mg/dl)	157.1 ± 94.1	174.7 ± 95.0	0.397
Albumin (G/dl)	4.24 ± 0.49	3.98 ± 0.56	0.007
ALT (U/L)	24.6 ± 56.6	19.4 ± 13.0	0.518
Alkaline phosphatase (U/L)	174.8 ± 116.3	177.4 ± 114.1	0.915
Sodium (meq/l)	134.09 ± 3.89	134.60 ± 4.74	0.551
Potassium (meq/l)	5.17 ± 0.84	5.12 ± 0.83	0.765
Calcium (mg/dl)	9.10 ± 0.96	9.03 ± 1.44	0.777
Phosphate (mg/dl)	4.92 ± 1.64	5.11 ± 1.76	0.577
Parathyroid hormone (pg/dl)	363.6 ± 230.8	342.1 ± 289.4	0.692

ALT: alanine transaminase. BMI: Body mass index. HD: haemodialysis. HDF: Hemodiafiltration. IDWG: interdialytic weight gain

### Factors associated with the use of antihypertensive agents

The 50th percentile (P50) was used to categorize the variables on a scale. The P50 values for the scale variables were as follows: IDWG, 4.925%; effective QB, 423.5 ml/min; transferrin saturation, 26.75%; glucose, 109.27 mg/dl; urea, 103.775 mg/dl; and albumin, 4.214 g/dl.

The risk factors for the use of antihypertensive agents were the presence of vascular amputation, a history of smoking, the presence of a diagnosis of type 2 diabetes mellitus, a % transferrin saturation > 26.75%, male sex, and hemodialysis as the treatment type.

A diagnosis of glomerulonephritis as the etiology of chronic renal failure, a history of never smoking, a serum ALB concentration > 4.216 g/dl, an effective Qb greater than 423.5 ml/min, an IDWG greater than 4.925%, hemodiafiltration as treatment, urea < 103.78 mg/dl, and fasting glucose < 109.27 mg/dl were identified as statistically significant protective factors (Table 2).

**Table 2:** Risk and protective factors for the use of antihypertensives

	Group 1 Without Antihypertensives *N* = 52	Group 2 With Antihypertensives *n* = 54	*P*	OR*	95% CI of the OR
**Risk factor’s**					
Legs vascular amputation	0 (0%)	9 (16.7%)	0.002	21.923	1.241-387.216
Ex-smoker	1 (1.9%)	10 (18.5%)	0.005	11.591	1.427–94.163
Type 2 Diabetes	19 (36.5%)	35 (64.8%)	0.003	3.199	1.446–7.078
Transferrin saturation > 26.75%	21 (40%)	35 (64.8%)	0.010	2.719	1.238–5.972
Male	18 (34.6%)	35 (64.8%)	0.002	1.842	1.224–2.772
Female	34 (65.4%)	19 (35.2%)			
Haemodialysis as treatment	5 (9.6%)	16 (29.6%)	0.009	1.704	1.218–2.385
**Protection factors**					
Glomerulonephritis as etiology of CKD	16 (30.7%)	5 (9.3%)	0.005	0.230	0.077–0.685
Smokin never	44 (84.6%)	31 (57.4%)	0.002	0.245	0.097–0.619
Albumin > 4.214 gr/dL	31 (59.6%)	19 (35.2%)	0.010	0.368	0.167–0.808
Effective QB > 423.5 ml/min	32 (61.5%)	21 (38.9%)	0.016	0.398	0.182–0.869
IDWG > 4.925%	32 (61.5%)	22 (40.7%)	0.026	0.430	0.197–0.936
Hemodiafiltration as treatment	47 (90.4%)	38 (70.4%)	0.009	0.431	0.196–0.947
Urea < 103.78 mg/dL	31 (59.6%)	21 (38.9%)	0.026	0.661	0.446–0.979
Glucose < 109.27 mg/dl	31 (59.6%)	19 (35.2%)	0.012	0.608	0.404–0.914
**Nonsignificant factors**					
Current-smoker	1 (1.9%)	0 (0%)	0.491	0.339	0.014–8.529
Hypertension as etiology of CKD	8 (15.4%)	6 (11.1%)	0.359	0.688	0.221–2.138
Polycystic Kidney disease	3 (5.8%)	2 (3.7%)	0.482	0.628	0.101–3.921
CKD of unknown etiology.	8 (15.4%)	7 (13.0%)	0.468	0.819	0.274–2.447
Access fistula	40 (76.9%)	39 (72.2%)	0.370	0.780	0.324–1.877
Access graft	4 (7.7%)	1 (1.91%)	0.170	0.226	0.024–2.097
Catheter	8 (15.4%)	14 (25.9%)	0.136	1.925	0.731–5.070
Diabetic blindness or significant retinal vascular injury	6 (11.5%)	14 (25.9%)	0.049	2.683	0.943–7.638
Cancer	1 (1.9%)	1 (1.9%)	0.743	0.962	0.059–15.798

*Odds ratio for the presence of the risk factor and the use of antihypertensive drugs

### Main results

#### Mortality and survival analysis

**Fig. 3:**
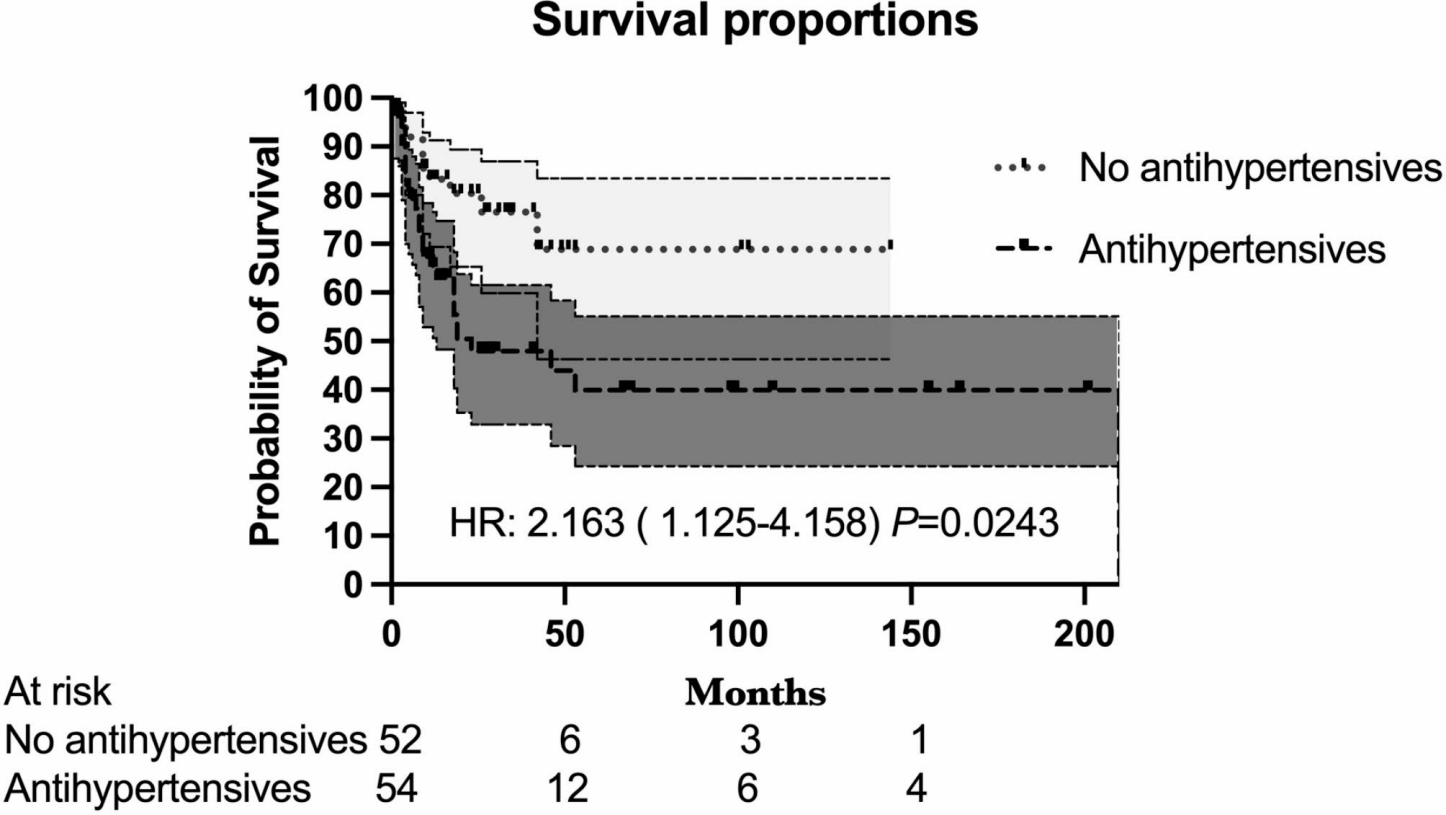
Survival proportions

The group that required antihypertensive agents had a significantly greater rate of mortality, with 11 patients (21.2%) compared to 25 patients (46.3%) in the other group (*P* = 0.005). On the other hand, group 1 had higher survival rates, with an HR of 2.2163 (1.125–4.158) and *P* = 0.0243, as shown in Fig. 3. The antihypertensive agent group (Group 2) had a survival time of 46 months, while Group 1 had indefinite survival, with a 70% probability of survival at 144 months at the end of the study.

#### Secondary analyses

**Fig. 4:**
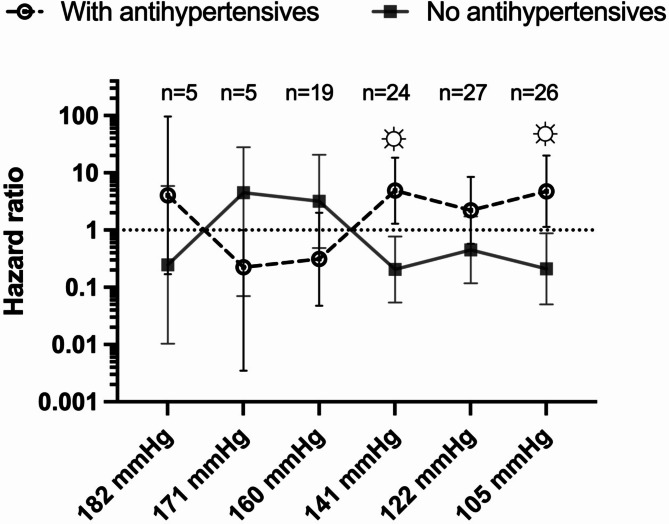
Hazard ratio according to sextile blood pressure

The Cox equation was used to predict the outcome of antihypertensive medication use based on survival (Table 3). The variables were significant: age, transferrin saturation, serum albumin concentration, and history of vascular amputation of a limb or part of the limb. According to the analysis stratified by sextile, a sextile less than 141 mmHg and greater than 122 mmHg predialysis systolic blood pressure and the use of antihypertensive agents increased the risk of death [HR 4.877 (1.297–18.34) *P* = 0.0141]. With a predialysis systolic blood pressure of less than 105 mmH6, antihypertensive therapy increased the risk of death [HR 4.764 (1.138–19.94) *P* = 0.010]. No other significant associations were found according to blood pressure level (Fig. 4).

**Table 3:** Variables in the COX equation

	B	SE	Wald	df	Sig.	Exp(B)	95.0% CI for Exp(B)		
							Lower	Upper	
Age (Years)	0.035	0.017	4.201	1	0.040	1.036	1.002	1.072	
Transferrin saturation (%)	-0.028	0.014	3.759	1	0.053	0.973	0.946	1.000	
Albumin (g/dl)	-2.093	0.398	27.692	1	< 0.001	0.123	0.057	0.269	
Vascular amputation of a leg or part of it	1.363	0.582	5.494	1	0.019	3.909	1.250	12.222	

-2 Log Likelihood 156.9, Chi-square 42.5, df 4, Sig < 0.001

## Discussion

### Main findings of the study

The main finding confirms the hypothesis of the study that there is more remarkable survival in the group of patients with CKD whose hypertension can be controlled without antihypertensive treatment and with the use of constant dry weight reduction measures to optimize ultrafiltration. The factors associated with the lack of control of arterial hypertension were a history of vascular amputation, a history of being an ex-smoker, being a carrier of type 2 diabetes mellitus, having a serum ferritin level greater than 26.75%, being male, and being treated with hemodialysis. The associated protective factors were having a diagnosis of glomerulonephritis as an etiology of chronic kidney disease, a history of never smoking, a serum albumin concentration greater than 4.214 g/dl, effective blood flow greater than 423.5 ml/min, and interdialytic weight gain > 4.925%, hemodiafiltration as treatment, urea levels less than 103.78 mg/dl, and fasting glucose levels less than 109.2 mg/dl. According to the time-adjusted model, only four factors were associated: age, transferrin saturation, serum albumin levels, and history of vascular amputation.

In the stratified analysis, differences in survival were demonstrated by the percentiles of blood pressure taken in the last month of survival or censoring. With blood pressures ranging from 141 mmHg to 122 mmHg, there is a proportional risk of death associated with the intake of antihypertensive agents. The same occurs when the blood pressure is less than 105 mmHg. These relationships could not be established with pressures greater than 141 mmHg.

### Importance of the findings

These findings are significant because they suggest that a lack of blood pressure control in patients with CKD undergoing hemodiafiltration or hemodialysis, despite optimization of dry weight, may be associated with poorer survival. Adequate blood pressure control is essential for reducing the risk of cardiovascular events, such as myocardial infarction, stroke, and death. In this study, blood pressure control with ultrafiltration was possible in 49.1% of patients, with a confidence interval ranging from 39.58 to 58.62%. This finding suggested that identifying and treating factors contributing to the lack of blood pressure control is essential. Some factors that may contribute to the lack of blood pressure control in patients with CKD include hypervolemia, malnutrition, excess transferrin saturation, and arteriolopathy, which can cause peripheral ischemia (vascular amputation).

### Studies with related findings

Similar observational studies have been reported previously [10, 11]; however, these studies do not consider the differentiation of higher or lower mortality among patients taking antihypertensive drugs. Specifically, in patients undergoing hemodialysis, the CONVINCE study published in 2023 revealed that patients receiving hemodiafiltration treatments had lower mortality than did those receiving hemodialysis (HR 0.77 CI 95% 0.65–0.93); however, the CONVINCE study did not distinguish patients receiving antihypertensive drugs in the sub-analysis of groups of patients receiving antihypertensive drugs [12]. Our group has presented a study on the lower incidence of intradialytic hypotension in patients in hemodiafiltration programs without studying mortality [13]. The present study is a continuation of this line of research. One clinical study of 126 hemodialysis patients was randomized into two groups: the first had a systolic pressure of 110–140 mmHg with intensive antihypertensive treatment, and the second had a systolic pressure of 155–165 mmHg with standard therapy for one year. There was no difference in mortality between the groups studied; there were four deaths in the intensive group versus 1 in the standard group (OR 4.34 95% CI 0.47–40) (*P* = 0.1947) [14]. A study of risk factors that do not allow the attainment of dry weight in transplanted patients revealed that the use of antihypertensive drugs, the use of a peritoneal dialysis program, and the presence of residual diuresis were the factors that contributed to a difference of more than 2 kg of the posttransplant dry weight to the estimated pretransplant dry weight [15].

### Alternative explanations

This study explicitly addresses hemodialysis patients whose blood pressure cannot be controlled with only ultrafiltration measures, diet, and water restriction but who require additional measures such as antihypertensive drugs. This group of patients with uncontrollable hypertension are food transgressors who are not limited in terms of sodium and fluid intake, patients with malnutrition and low oncotic pressure, and patients with arteriolopathies. With clinical evaluation at the bedside, an attempt has been made to exclude hypovolemic patients who unnecessarily take antihypertensive drugs from the group. The study used an active ultrafiltration strategy for eight weeks and intradialytic monitoring by a nephrologist who conducted ultrafiltration throughout the treatment. This ultrafiltration maneuver, called POC-DW, clearly differentiates the two groups and presents the risk factors that do not allow us to reach a pre- and post-dialysis systolic blood pressure below the target range of the study. Groups 1 and 2 are not comparable; instead, they are intended to perform a statistical contrast, which may be one of the reasons why the results are inclined toward Group B (without antihypertensive drugs). One example of this contrast is age; there is an age difference of 9 years between Group 1 and Group 2, which is statistically significant. Age in itself constitutes a mortality factor in patients with chronic kidney disease. On the other hand, the mean systolic pressure of Group 2 was 143 mmHg, which was six mmHg higher than that of Group 1 because it was the classification factor of the groups.

### Clinical relevance of the findings

This study provides a methodology for continuous ultrafiltration with point-of-care dry weight. Its novelty is that active ultrafiltration measures may be sufficient for some patients and could affect current treatment paradigms. This methodology can begin an artificial intelligence algorithm for automated dry-weight programming. Additionally, hemodiafiltration treatment must have a clinically effective replacement volume (> 22 L) and an extracorporeal flow prescription good enough to provide adequate clearance (Qb greater than 423.5 ml/min).

### Limitations of the study

The hemodiafiltration unit where the study was carried out regularly attends to 100 cases in the Amazon region of Ecuador out of a population of 192,505 inhabitants (519 cases per million inhabitants), where all the cases of the 4-year observation period were included. In retrospect, the statistical power of the sample size was calculated with Epi info™ 7.2 (CDC, Office of Public Health Data, Surveillance, and Technology, November 2021, Atlanta, USA), representing a confidence level of 80%. With a confidence limit of 5%, an expected frequency of 20.9%, and a population size of 3052 cases for Morona Santiago province and Guayaquil in Ecuador. Because of the small sample size, multicenter studies are needed to validate our results. The follow-up period of approximately four years may not be sufficient to observe the long-term results and complications associated with blood pressure control in a small cohort of CKD patients.

Another limitation was that the bioimpedance assessment was performed in only some of the cases presented, so these results were omitted.

### Future research

Future studies should address the dry weight obtained by impedance and its long-term relationship with the use of antihypertensives. Additionally, in patients who cannot reach dry weight without using antihypertensive drugs, several different ultrafiltration maneuvers should be randomized and established to design the best long-term ultrafiltration treatment and reduce mortality.

### Main messages

- Ultrafiltration can only control arterial hypertension in a large group of patients (49.1%).
- Lack of blood pressure control is a significant risk factor for mortality in patients with chronic kidney disease who undergo hemodialysis or hemodiafiltration.
- CKD patients who require the use of antihypertensives have a greater risk of mortality than CKD patients who do not require the use of antihypertensives.
- Identifying and treating the factors contributing to poor blood pressure control in CKD patients with Point of Care Dry Weight (POC-DW).

## Conclusions

The findings of this study suggest that blood pressure control with active ultrafiltration measures and without the use of antihypertensive agents is an essential factor that contributes to more remarkable survival in patients with CKD in hemodiafiltration and hemodialysis programs. The use of antihypertensive drugs in patients on hemodiafiltration and hemodialysis programs, with pressures between 141 and 122 mmHg and less than 105 mmHg, can be harmful.

## Electronic supplementary material

Below is the link to the electronic supplementary material.

Supplementary Material 1

## Data Availability

Data is provided within the manuscript or supplementary information files.

## Abbreviations

CKD: Chronic kidney disease
HR: Hazard ratio
POCDW: Point of Care Dry Weight
